# Transcriptome profiling-based identification of prognostic subtypes and multi-omics signatures of glioblastoma

**DOI:** 10.1038/s41598-019-47066-y

**Published:** 2019-07-22

**Authors:** Junseong Park, Jin-Kyoung Shim, Seon-Jin Yoon, Se Hoon Kim, Jong Hee Chang, Seok-Gu Kang

**Affiliations:** 1 Department of Neurosurgery, Brain Tumor Center, Severance Hospital, Yonsei University College of Medicine, Seoul, Republic of Korea; 2 Department of Biochemistry and Molecular Biology, Yonsei University College of Medicine, Seoul, Republic of Korea; 3 Department of Pathology, Severance Hospital, Yonsei University College of Medicine, Seoul, Republic of Korea

**Keywords:** CNS cancer, Cancer genomics, Classification and taxonomy, Microarrays

## Abstract

Glioblastoma (GBM) is a lethal tumor, but few biomarkers and molecular subtypes predicting prognosis are available. This study was aimed to identify prognostic subtypes and multi-omics signatures for GBM. Using oncopression and TCGA-GBM datasets, we identified 80 genes most associated with GBM prognosis using correlations between gene expression levels and overall survival of patients. The prognostic score of each sample was calculated using these genes, followed by assigning three prognostic subtypes. This classification was validated in two independent datasets (REMBRANDT and Severance). Functional annotation revealed that invasion- and cell cycle-related gene sets were enriched in poor and favorable group, respectively. The three GBM subtypes were therefore named invasive (poor), mitotic (favorable), and intermediate. Interestingly, invasive subtype showed increased invasiveness, and *MGMT* methylation was enriched in mitotic subtype, indicating need for different therapeutic strategies according to prognostic subtypes. For clinical convenience, we also identified genes that best distinguished the invasive and mitotic subtypes. Immunohistochemical staining showed that markedly higher expression of PDPN in invasive subtype and of TMEM100 in mitotic subtype (*P* < 0.001). We expect that this transcriptome-based classification, with multi-omics signatures and biomarkers, can improve molecular understanding of GBM, ultimately leading to precise stratification of patients for therapeutic interventions.

## Introduction

Glioblastoma (GBM) is one of the most feared human diseases due to high mortality rate and accompanying loss of cognitive function during the disease process. At present, however, there are few prognostic biomarkers and predictors of therapeutic response, as well as few therapeutic interventions strongly affecting disease outcome^1–3^. Although patients with *IDH1* pathogenic variants have a significantly better prognosis than those with wild-type *IDH1*, these pathogenic variants are observed in only 4–7% of primary GBM patients, restricting their use as a biomarker^4^. Similarly, DNA methylation status in *MGMT* promoter region is a predictive biomarker for response to temozolomide treatment^5^, but it is applicable only to the non-recurrent classical subtype GBM^6^.

Cancers are being increasingly classified based on their histopathological and molecular characteristics, leading to the trend of precision cancer medicine^7–9^. GBM has been classified into several molecular subtypes based on their gene expression profiles: classical, mesenchymal, (neural), and proneural^1,10^. Although these subtypes have distinct molecular signatures and etiologic factors, their relationship with overall survival (OS) is ambiguous except for patients with *IDH1* pathogenic variant^1,10,11^. The inability to determine patient outcomes based on histopathological features and current molecular subtypes inhibits the ability to effectively manage GBM. Thus, clinically relevant GBM subtypes, with sufficient information on prognosis and biological phenotype, are required to optimize treatment. In this regard, this study assessed the ability of novel transcriptome-based prognostic subtypes and their molecular multi-omics signatures for new taxonomy of GBM patients: invasive, mitotic, and intermediate. We expect that our prognostic stratification of GBM has important clinical implications for diagnosis and treatment of patients, providing a framework that unifies transcriptomic, genomic, and clinical signatures.

## Results

### Overview of the approach

**Figure 1:**
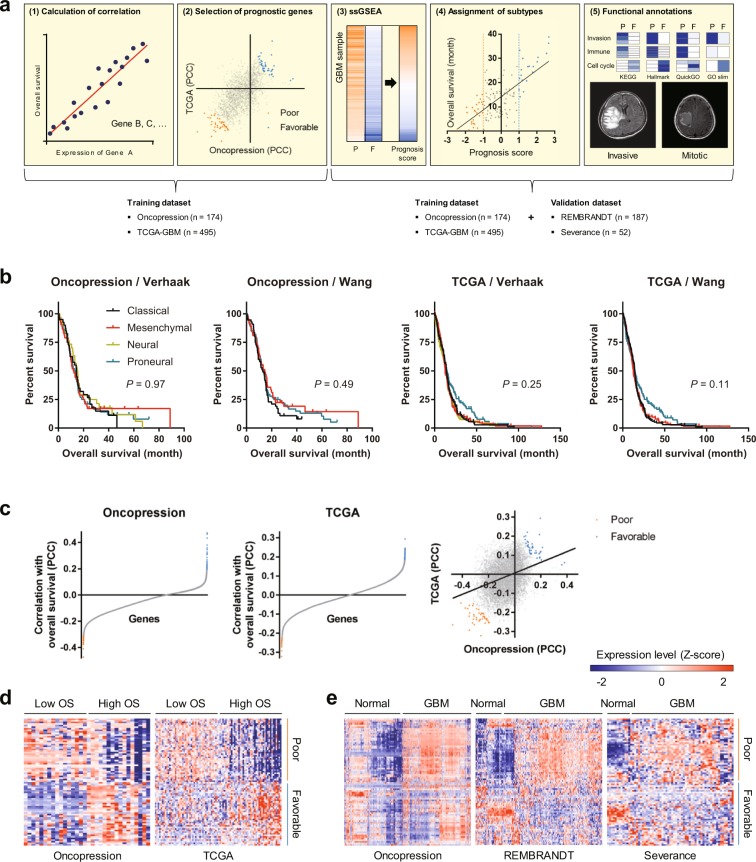
Flow chart of the study and selection of PGs. (**a**) Graphical flow chart describing the calculation, assignment, and functional annotation of GBM prognostic subtypes. (**b**) GBM datasets retrieved from oncopression and TCGA databases were assorted by Verhaak’s molecular subtypes, and OS was compared by the Kaplan-Meier method (not significant by log-rank test). (**c**) Each dot indicates PCC of each gene calculated by correlation with OS. In the right panel, PCCs calculated by oncopression (x-axis) and TCGA-GBM (y-axis) are presented as a scatter plot with a linear regression line (*P* < 0.001). The 40 highest (favorable) and lowest (poor) genes (PGs) are marked with colors. (**d**,**e**) Expression levels of these 80 PGs were compared in patients with low OS and high OS (**d**), and between normal and GBM samples (**e**).

Figure 1a shows a graphic flow chart of this study: (1) The correlation between expression level of each gene and patient OS was calculated using oncopression and the cancer genome atlas (TCGA)-GBM databases. (2) Forty genes correlating with poor prognosis and 40 correlating with favorable prognosis (PGs) were selected. (3–4) GBM samples were subjected to single sample gene set enrichment analysis (ssGSEA) using these PGs, and prognostic subtypes were assigned. In addition to oncopression and TCGA-GBM, repository for molecular brain neoplasia data (REMBRANDT) and Severance datasets were used for validation. (5) Prognostic subtypes were functionally annotated using over-representation analysis (ORA); thereby, subtypes with poor and favorable prognosis were named “invasive” and “mitotic”, respectively.

### Identification of PGs in GBM using transcriptome analysis

GBM samples were classified according to Verhaak’s subtypes, the most widely used gene expression-based classification of GBM^1,10^, and OS was compared in these subtypes by the Kaplan-Meier method. Although several distinct molecular features were reported, there was no relationship between these subtypes and OS (Fig. 1b). To identify PGs, Pearson’s correlation coefficients (PCCs) were calculated between the expression level of each gene and patient OS. Among them, genes having the highest PCCs in both oncopression and TCGA-GBM were classified as PGs (Fig. 1c and Table 1). Supplementary Fig. S1 shows functional interactions among these PG sets. As expected, the 40 poor PGs showed higher expression levels in patients with shorter OS, whereas the 40 favorable PGs showed higher expression levels in patients with longer OS (Fig. 1d). Notably, most poor PGs showed higher expression in GBM than in normal samples (Fig. 1e).

**Table 1:** Gene sets associated with prognosis of GBM patients (PGs).

Poor (Invasive)				Favorable (Mitotic)			
Entrez	Symbol	Entrez	Symbol	Entrez	Symbol	Entrez	Symbol
4478	MSN	5269	SERPINB6	10202	DHRS2	338645	LUZP2
6990	DYNLT3	1819	DRG2	3929	LBP	4093	SMAD9
3964	LGALS8	873	CBR1	3250	HPR	10566	AKAP3
6281	S100A10	9737	GPRASP1	55273	TMEM100	3484	IGFBP1
3958	LGALS3	10630	PDPN	55506	H2AFY2	5994	RFXAP
9208	LRRFIP1	51150	SDF4	79097	TRIM48	5449	POU1F1
1635	DCTD	23150	FRMD4B	9934	P2RY14	9472	AKAP6
64114	TMBIM1	2934	GSN	55176	SEC. 61A2	92211	CDHR1
2014	EMP3	19	ABCA1	1602	DACH1	5978	REST
9516	LITAF	9948	WDR1	2660	MSTN	6898	TAT
7037	TFRC	54431	DNAJC10	8366	HIST1H4B	1656	DDX6
1534	CYB561	4016	LOXL1	1007	CDH9	10214	SSX3
30008	EFEMP2	30836	DNTTIP2	51079	NDUFA13	7783	ZP2
2152	F3	301	ANXA1	2674	GFRA1	2153	F5
57212	TP73-AS1	23351	KHNYN	10683	DLL3	6445	SGCG
10723	SLC12A7	10123	ARL4C	11077	HSF2BP	9906	SLC35E2
8996	NOL3	7110	TMF1	8854	ALDH1A2	27296	TP53TG5
5352	PLOD2	10404	CPQ	1360	CPB1	79955	PDZD7
114883	OSBPL9	9077	DIRAS3	79896	THNSL1	79727	LIN28A
9325	TRIP4	6282	S100A11	3567	IL5	266	AMELY

### Assignment of prognostic subtypes for GBM

**Figure 2:**
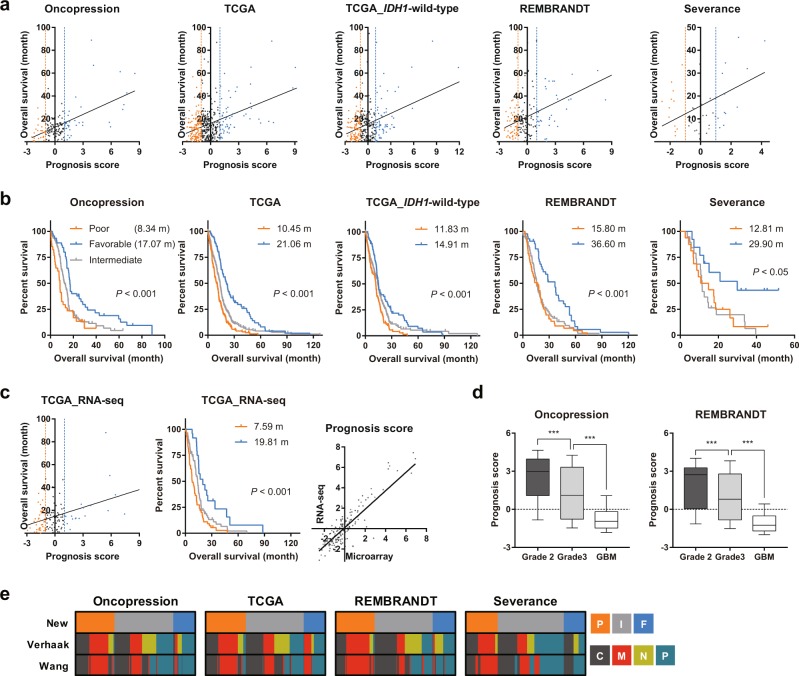
Assignment of prognostic subtypes. (**a**) Each dot indicates the prognosis score and OS of each GBM sample. Vertical dashed lines indicate threshold values (−1 and 1) for subtype assignment. The Pearson correlation was significant in all datasets; the linear regression line is shown in black. (**b**) Survival probability for each prognostic subtype was estimated based on Kaplan-Meier curves. Statistical significance was determined by the log-rank test (*P* < 0.001 for oncopression, TCGA-GBM, and REMBRANDT, *P* < 0.05 for Severance). (**c**) Corresponding presentation with (**a**) and (**b**) (left and center, respectively) was shown using RNA-seq data (TCGA). Scatter plot shows the correlation between prognosis scores of the matched patients obtained from microarray and RNA-seq data (TCGA; right). The Pearson correlation was significant (*P* < 0.001, *R* = 0.84); the linear regression line is shown in black. (**d**) Prognosis scores were compared among grades 2–4 glioma samples from oncopression and REMBRANDT. Differences among groups were compared by one-way ANOVA with Tukey’s *post hoc* test for multiple comparisons; ***P < 0.001. (**e**) Distribution of GBM molecular subtypes is presented as heat maps. Upper line: P, poor; I, intermediate; F, favorable. Middle and bottom lines: C, classical; M, mesenchymal; N, neural; P, proneural.

Using these PGs, GBM samples were subjected to ssGSEA to evaluate their prognosis scores, the criterion for subtype assignment; patients with prognosis scores <−1, >1, and between −1 and 1 were classified into the poor (invasive), favorable (mitotic), and intermediate subtypes, respectively (Supplementary Fig. S2). Linear regression analyses of four independent datasets – oncopression, TCGA-GBM, REMBRANDT, and Severance datasets – revealed that prognosis score correlated significantly with OS of GBM patients (Fig. 2a). Cox regression using prognosis score as univariate provides hazard ratios (HRs) with 95% confidence intervals (CIs): oncopression, HR = 0.837 (0.770–0.910); TCGA-GBM, HR = 0.804 (0.736–0.878); REMBRANDT, HR = 0.818 (0.738–0.907); Severance, HR = 0.780 (0.650–0.934). Each of the GBM datasets was divided into three groups according to their prognosis scores, and OS was compared in these groups using the Kaplan-Meier method. In all datasets, OS was significantly longer in the favorable than in the poor group, confirming that this transcriptome-based GBM classification into prognostic subtypes reflects patient OS (Fig. 2b and Supplementary Table S1). Notably, TCGA-GBM samples including only *IDH1*-wild-type patients – distinguished with *IDH1*-mutant GBM in WHO classification of tumors 2016 – also showed similar patterns, suggesting that longer OS of favorable group did not simply arise from *IDH1* pathogenic variant (Fig. 2a,b). In addition, similar patterns were observed in RNA-seq data (TCGA), and the prognosis scores of the matched patients in these two platforms showed significant correlation, suggesting that this method is applicable to both microarray and RNA-seq platforms (Fig. 2c). We also evaluated prognosis scores in low-grade glioma samples (grade 2 and 3). Prognosis scores decreased significantly with increasing tumor grade, suggesting that this method is applicable to datasets that include low-grade glioma samples (Fig. 2d). When we examined the relationship of this classification with Verhaak’s molecular subtypes, we found that the mesenchymal subtype was more enriched in the poor than in the favorable group, whereas the proneural subtype was more enriched in the favorable than in the poor group (Fig. 2e).

### Functional annotation of GBM prognostic subtypes

**Figure 3:**
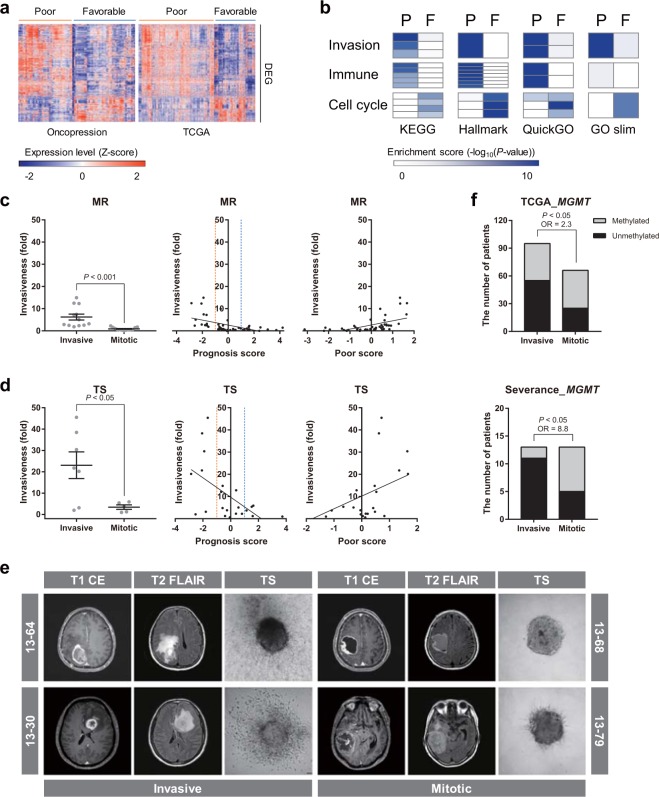
Functional annotation to prognostic subtypes. (**a**) Expression levels of DEGs were displayed as a heat map. DEGs were defined as genes with *P* < 0.001 (FDR correction for multiple comparisons) between poor and favorable groups. (**b**) Functional annotation of DEGs was performed by ORA. Statistical significance was determined using Fisher’s exact test, and enrichment scores are presented as a heat map. P, poor; F, favorable. (**c**–**e**) Using Severance dataset, invasiveness was evaluated in MR images of GBM patients (**c**) and GBM TSs (**d**). Scatter plots show correlation between invasiveness and prognosis or poor score. Representative figures are presented in (**e**). (**f**) DNA methylation status in *MGMT* promoter region was compared between prognostic subtypes (Severance; OR = odds ratio). Differences in subtypes were compared by two-tailed Student’s *t*-test in (**a**,**c**,**d**) and by Fisher’s exact test in (**f**).

To determine the biological characteristics of each prognostic subtype, we first identified differentially expressed genes (DEGs) between poor and favorable groups in oncopression and TCGA-GBM datasets (Fig. 3a and Supplementary Data S1). These DEGs were then subjected to ORA for functional annotation. ORA using four gene set databases revealed that invasion- and immune-related gene sets were significantly enriched in the poor group, whereas cell cycle-related gene sets were significantly enriched in the favorable group (Fig. 3b). Similar results were reproduced in enrichment maps using gene ontology (GO) hierarchy, in that many enriched GO terms in the poor group were in cell migration and invasion modules, whereas cell cycle-related GO terms were enriched in the favorable group (Supplementary Fig. S3). Because signal transduction- and immune-related gene sets are frequently enriched in non-tumor samples, we focused on invasion-related gene sets in the poor group. The poor group was therefore named the “invasive” subtype, and the favorable group was named the “mitotic” subtype. Interestingly, prognosis scores significantly correlated with the invasive property of GBM samples, as illustrated in both MR images of GBM patients (Fig. 3c) and collagen-based *in vitro* 3D invasion assays of patient-derived GBM tumorspheres (TSs; Fig. 3d). Representative images of both subtypes are presented in Fig. 3e. In addition, *MGMT* methylation was significantly enriched in the mitotic subtype, indicating that different therapeutic strategies are required in treating patients with these two prognostic subtypes (Fig. 3f). Ki-67 expression did not differ significantly in the two subtypes, probably because proliferation is a universal hallmark of cancer. The clinical characteristics of patients, including age and sex, did not affect subtype classification (Supplementary Fig. S4). HRs with 95% CIs obtained by Cox regression model were provided in Supplementary Table S2. Collectively, these data suggest that prognostic subtypes have distinct biological phenotypes, differing especially in invasive properties.

### Genomic signatures of GBM prognostic subtypes

**Figure 4:**
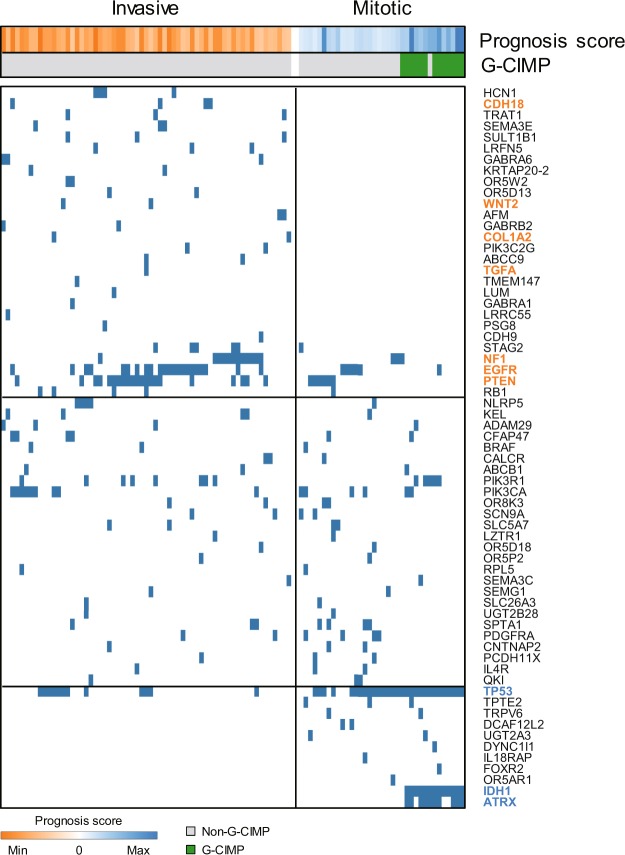
Genomic signatures of prognostic subtypes (TCGA-GBM). Distribution of somatic pathogenic variants of recurrently altered genes in GBM. Samples were separately clustered according to prognostic subtypes, and heat maps indicate prognosis score or G-CIMP status of each sample. Invasive or mitotic subtype samples with at least one pathogenic variant were included. Genes mentioned in the text are highlighted.

We next examined multi-omics signatures of each prognostic subtype. The distribution of genomic alterations in recurrently mutated genes (TCGA) showed that pathogenic variants in several genes occurred exclusively in the invasive or mitotic subtype. Pathogenic variants in *CDH18*, *WNT2*, *COL1A2*, and *TGFA*, all of which are associated with invasion^12–14^, were observed only in the invasive subtype. In contrast, pathogenic variants in *IDH1* and *ATRX*, which are associated with good prognosis^4^, were observed only in the mitotic subtype, consistent with our prognostic subtype classification. Moreover, glioma-CpG island methylator phenotype (G-CIMP), which is associated with good prognosis^15^, was exclusively observed in the mitotic subtype (Fig. 4). DNA methylation status other than G-CIMP also showed distinct patterns in the invasive and mitotic subtypes (Supplementary Fig. S5). Differences in copy number alteration (CNA) and corresponding gene list were also demonstrated in Supplementary Fig. S5 and Supplementary Data S2.

### Markers of GBM prognostic subtypes

**Figure 5:**
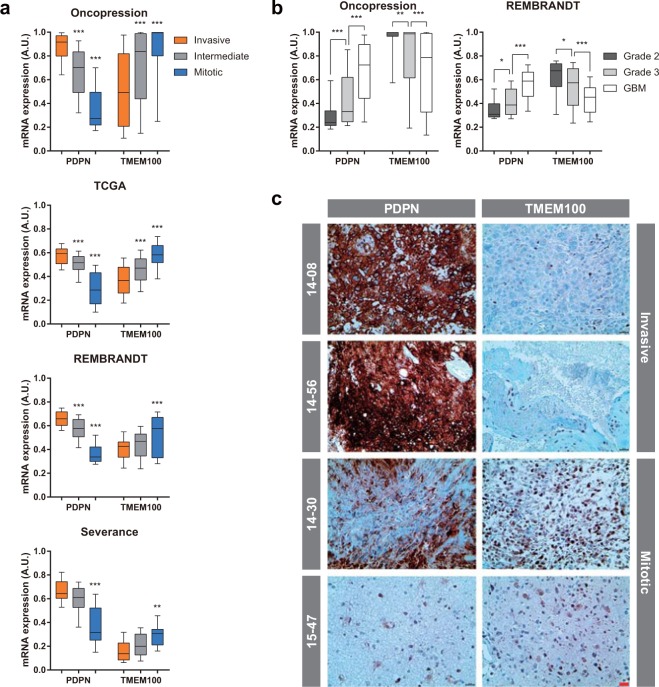
Expression of markers for prognostic subtypes. (**a**,**b**) Expression levels of *PDPN* and *TMEM100* in each prognostic subtype (**a**) and in grades 2–4 glioma (**b**). Differences among groups were compared by one-way ANOVA with Tukey’s *post hoc* test for multiple comparisons; *P < 0.05, **P < 0.01, ***P < 0.001 in (**a**) denote significant differences compared with the invasive group. (**c**) Expression levels of PDPN and TMEM100 were measured by IHC (brown). In all images, hematoxylin (blue) was used to counterstain nuclei (red scale bar = 20 μm).

For clinical convenience, we also identified the genes that best differentiated between the invasive and mitotic subtypes, based on the intersection between PGs listed in Table 1 and the DEGs identified in Fig. 3a. Of the 48 genes identified, *PDPN* and *TMEM100* showed the greatest differential expression between these two prognostic subtypes, except for genes encoding secreted proteins (Supplementary Data S1). In all four independent datasets, *PDPN* showed significantly higher expression levels in the invasive subtype, whereas *TMEM100* showed significantly greater expression in the mitotic subtype (Fig. 5a). Moreover, expression levels of *PDPN* were significantly correlated with increasing glioma grade, whereas *TMEM100* showed the opposite pattern (Fig. 5b), suggesting that both of these markers are associated with prognosis, even when low-grade glioma samples were included. Immunohistochemistry (IHC) confirmed markedly higher PDPN levels in the invasive subtype and TMEM100 levels in the mitotic subtype (Fig. 5c). These findings suggest that PDPN is a marker for the invasive subtype, and TMEM100 is a marker for the mitotic subtype.

## Discussion

Owing to enormous heterogeneity of tumors including GBM^16^, histologically defined tumors should be further divided into subgroups using molecule-level criteria. Categorizing GBM into subtypes may result in more precise treatment, enabling rational therapy based on subgroup-specific targets. Here, we show prognostic subtypes of GBM in terms of large-scale gene expression profiles. These novel GBM subtypes had differential biological phenotypes and multi-omics signatures, including differences in somatic pathogenic variants, DNA methylation, and CNA. The reproducibility of this classification was validated in four independent datasets, including one based on samples from our institution (Severance).

The importance of this study lies in that these prognostic subtypes are interrelated with distinct biological phenotypes, such as invasiveness. Migratory and invasive capabilities of tumor, along with mesenchymal transition and distant metastasis, are hallmarks of cancer associated with poor prognosis^17,18^. Of our prognostic subtypes of GBM, the poor (invasive) subtype showed significantly greater invasiveness than the favorable (mitotic) subtype, consistent with previous findings. Moreover, a methylated *MGMT* promoter region correlated significantly with the mitotic subtype, indicating that patients with this subtype were more likely to respond to temozolomide^5^. These prognostic subtypes are clinically relevant, resulting in patient stratification and enhancing integrative understanding of GBM. These results also suggest that therapeutic strategies should be based on prognostic subtypes; for example, patients with the mitotic subtype can be treated with temozolomide, whereas patients with the invasive subtype should receive therapeutic interventions targeting tumor invasiveness. In this regard, we will evaluate novel therapeutic strategy targeting invasiveness in the future study.

The prognostic subtypes identified here are in good agreement with previously reported GBM prognosis-associated signatures, including *IDH1* pathogenic variant and G-CIMP. Although prognostic subtypes were based solely on transcriptome and OS, genomic signatures such as somatic pathogenic variants in *IDH1* and *ATRX*, and G-CIMP, a DNA methylation signature, were exclusively present in the mitotic subtype. Because these signatures have been associated with favorable outcomes^4,15^, our subtypes reflect not only transcriptomic factors but also previously reported multi-omics markers in GBM patients. Interestingly, several genes in poor PG set were overlapped with previously reported G-CIMP transcriptome signature genes^19^. Studies are needed to determine whether this sample subset can be identified using both our OS-based subtypes and somatic pathogenic variant- or methylation-based subtypes. Moreover, our method was validated in TCGA RNA-seq data as well as in various microarray chips, implying its flexibility for multi-platform analyses.

Classification based on transcriptomes and OS has a distinct advantage, as in practice it is difficult to obtain all available multi-omics data, including transcriptome, genome, methylome, and DNA structural variation, from individual patients due to cost and overtreatment problems. Because our method uses only transcriptome and OS information, the prognostic subtypes we identified may be applicable clinically, as well as in research. Moreover, we identified single gene markers for each prognostic subtype, diminishing costs per patient, even though these results may not be as robust as those obtained from genome-wide expression levels. Future biomarker assays for GBM may include molecular tests for these prognostic subtypes.

Future studies are also required to assess the list of poor PGs (Table 1). In addition to determining prognostic subtypes, this list contains genes not previously recognized as GBM-associated genes. Because their expression levels are indicative of GBM prognosis, inhibition of subsets of these genes may prolong OS. Although a mechanistic explanation of all these genes in relevance to GBM progression is beyond the scope of this study, they may have clinical use as prognostic biomarkers and novel drug targets, as well as suggesting new insights into GBM pathology and etiologies.

## Methods

### Public datasets

The primary sources of samples were the oncopression^20^, TCGA, and REMBRANDT^21^ databases. From oncopression (http://oncopression.com), preprocessed gene expression data using microarray were retrieved (normal brain, n = 723; grade 2 astrocytoma, n = 133; grade 3 astrocytoma, n = 132; GBM, n = 865) and survival information was obtained for 174 GBM patients. From TCGA, preprocessed multi-omics GBM datasets were obtained through cBioPortal^22,23^ (U133 microarray, n = 495; RNA-seq, n = 166; somatic pathogenic variant data from whole exome sequencing, n = 491; methylation, n = 254 for HM27 and n = 84 for HM450; CNA from GISTIC 2.0, n = 478) with survival information of 496 GBM patients. Secondary or recurrent GBM samples were excluded, and G-CIMP status was determined as described^6^. The REMBRANDT gene expression dataset (E-MTAB-3073) was obtained from ArrayExpress (grade 2 astrocytoma, n = 65; grade 3 astrocytoma, n = 58; GBM, n = 228) with survival information of 187 GBM patients.

### Patient information (severance)

Samples were obtained from 52 non-recurrent GBM patients treated at Severance Hospital (Table 2). To obtain gene expression profiles using microarrays, total RNA was extracted from each tissue sample using Qiagen RNeasy Plus Mini kits, and loaded onto Illumina HumanHT-12 v4 Expression BeadChip (Illumina, San Diego, CA, USA). The data were variance stabilizing transformed and quantile normalized using the R/Bioconductor lumi package^24^. MR images of patients were taken using Achieva 3.0T system (Philips Medical Systems, Best, Netherlands) within 7 days before the respective brain tumor removal. Axial images were planned parallel to the anterior and posterior limb of the corpus callosum. Because T1 contrast enhanced (CE) and T2 fluid-attenuated inversion recovery (FLAIR) images nearly indicate primary tumor core region and invasive front region, respectively, we quantified invasiveness according to the equation – the area occupied by (T2 FLAIR - T1 CE)/T1 CE, as previously suggested^25^. Among whole axial-axis MR images, sections having the largest tumor area were selected for quantification of invasiveness, and two authors (J.P. and S.-J.Y.) independently measured tumor area to minimize rater bias. Experiments in this study were approved by the institutional review board of Severance Hospital, Yonsei University College of Medicine (4-2012-0212, 4-2014-0649), and all participants provided written informed consent. All experiments were performed in accordance with relevant guidelines and regulations.

**Table 2:** Clinical characteristics of the samples in Severance dataset.

Feature	Invasive (n = 14)	Mitotic (n = 14)	Intermediate (n = 24)
Age: Median (LQ-HQ)	56.5 (52–59.5)	60.5 (50.8–65.3)	60.5 (51.5–67)
Survival: Median (CI)	12.8 (10.2–15.4)	20.9 (16.8–24.9)	11.4 (9.2–13.7)
Sex: M/F	10/4	7/7	17/7
*IDH1*: Wild/Mut/Unknown	12/0/2	11/2/1	22/0/2
*MGMT* promoter: Met/Unmet/Unknown	2/11/1	8/5/1	7/17/0

### Isolation of GBM TSs and 3D invasion assay

TS-forming GBM cells were established from fresh GBM tissue specimens as previously described^26^. For TS culture^27^, cells were cultured in TS complete media, composed of DMEM/F-12 (Mediatech, Manassas, VA, USA), 1× B27 (Invitrogen, San Diego, CA, USA), 20 ng/mL bFGF, and 20 ng/mL EGF (Sigma-Aldrich, St. Louis, MO, USA). For 3D invasion assays^27^, each well of a 96-well plate was filled with mixed matrix composed of Matrigel, collagen type I (Corning Incorporated, Corning, NY, USA), and TS complete media. Single spheroids were seeded inside the matrix prior to gelation, followed by the addition of TS complete media over the gelled matrix to prevent drying. Invaded area was quantified as occupied area at (72 h–0 h)/0 h.

### Selection of prognosis-associated genes (PGs) and calculation of prognosis score

PGs were defined as genes highly correlated with OS of GBM patients in both oncopression and TCGA datasets. Genes whose PCCs were negative in both datasets were considered poor genes; genes whose PCCs were positive in both datasets were considered as favorable genes; and genes whose PCCs had different signs in these two datasets were excluded because they were associated with poor prognosis in one dataset and favorable prognosis in the other dataset. The product of PCCs with sign from these two datasets (PCC score) was regarded as a quantification of robust correlation (Supplementary Table S3). Because rare genes are significantly correlated with favorable prognosis, it is impractical to define PG sets larger than 40 genes for each prognostic subtype. After sorting according to this metric, therefore, 40 poor PGs and 40 favorable PGs were selected (Table 1). Using these PGs, GBM expression profiles were applied to ssGSEA, and enrichment scores were standardized across all samples. Poor and favorable scores of each GBM sample were defined as this standardized score, and prognosis score is defined as (favorable score - poor score). To confirm that the number of genes in each PG set is appropriate, we also calculated prognosis scores using top 20 × 2 genes rather than using 40 × 2 genes. Correlation between prognosis scores obtained using these two gene sets were statistically significant (*P* < 0.001 for all datasets), suggesting that the results were very similar (Supplementary Fig. S2). If the outcomes are similar, larger gene sets can generate robust result across independent cohorts. Based on these results, we finally defined two PG sets including 40 genes each. Functional interactions among PGs were constructed as network maps using Cytoscape^28^ and Reactome FI^29^ plug-in.

### Cutoff values of prognostic score and assignment of prognostic subtypes

Because TCGA-GBM dataset is not a validation set and has sufficient number of samples over various prognosis scores, we performed sensitivity analysis regarding several cutoff values for subtype assignment ranging from −2.0 to 2.0 using TCGA-GBM dataset. After dividing whole cohort into poor and favorable subgroups using specific cutoff value of prognosis score, we calculated *P*-values of log-rank test. This data shows that −1.0 indicate distinct local minimum *P*-value, suggesting −1.0 as one of good cutoff value (Supplementary Fig. S2). To preclude samples with moderate prognosis scores from being assigned into poor or favorable subtypes, we introduced intermediate subtype between poor and favorable subtypes, indicating necessity of one more cutoff value. Cutoff value of 1.0 generated very low *P*-value, shaping plateau pattern at cutoff values larger than 1.0. Because too inclined cutoff values cause large difference in subtype size (Supplementary Fig. S2), we finally selected −1.0 and 1.0 as cutoff values for assignment of prognostic subtypes.

### Functional annotation of prognostic subtypes

Functional annotation to DEGs between invasive and mitotic subtypes was performed by ORA using gene sets obtained from MSigDB (KEGG and hallmark), QuickGO, and GO slim databases. Gene sets were manually categorized according to the functional similarity of terms. Statistical significance was determined using Fisher’s exact test, and enrichment scores were depicted as a heat map (GENE-E software). Additionally, ORA results with GO terms were visualized as an enrichment map using Cytoscape and ClueGO^30^ plug-in. Enriched GO terms were functionally categorized based on their kappa scores (>0.4). Statistical significance was determined using two-sided hypergeometric test, and only nodes with Bonferroni-adjusted *P*-value < 0.001 were displayed.

### IHC of marker proteins

Brain tissues from GBM patients were sliced into 5-μm-thick sections using a microtome, and then transferred onto adhesive slides. Antigen retrieval and antibody attachment were performed using an automated instrument (Discovery XT, Ventana Medical Systems, Tucson, AZ, USA). PDPN (Santa Cruz Biotechnology, Santa Cruz, CA, USA) and TMEM100 (OriGene, Rockville, MD, USA) were detected using a peroxidase/DAB staining system. All images were counterstained with hematoxylin.

## Supplementary information

Supplementary Information

Supplementary Data S1

Supplementary Data S2

## Data Availability

The dataset (Severance) analysed during the current study is available in the GEO repository with accession number of GSE131837.
