# Supplementary material for: Transcriptome profiling-based identification of prognostic subtypes and multi-omics signatures of glioblastoma
Source: Sci Rep. 2019 Jul 22;9:10555. doi: 10.1038/s41598-019-47066-y (PMC6646357; doi:10.1038/s41598-019-47066-y)
Supplement: Supplementary file 1 — Supplementary Information [file 41598_2019_47066_MOESM1_ESM.pdf]

Transcriptome profiling-based identification of prognostic subtypes and multi-omics signatures of glioblastoma

Junseong Park, Jin-Kyoung Shim, Seon-Jin Yoon, Se Hoon Kim, Jong Hee Chang, Seok-Gu Kang

Supplementary Information

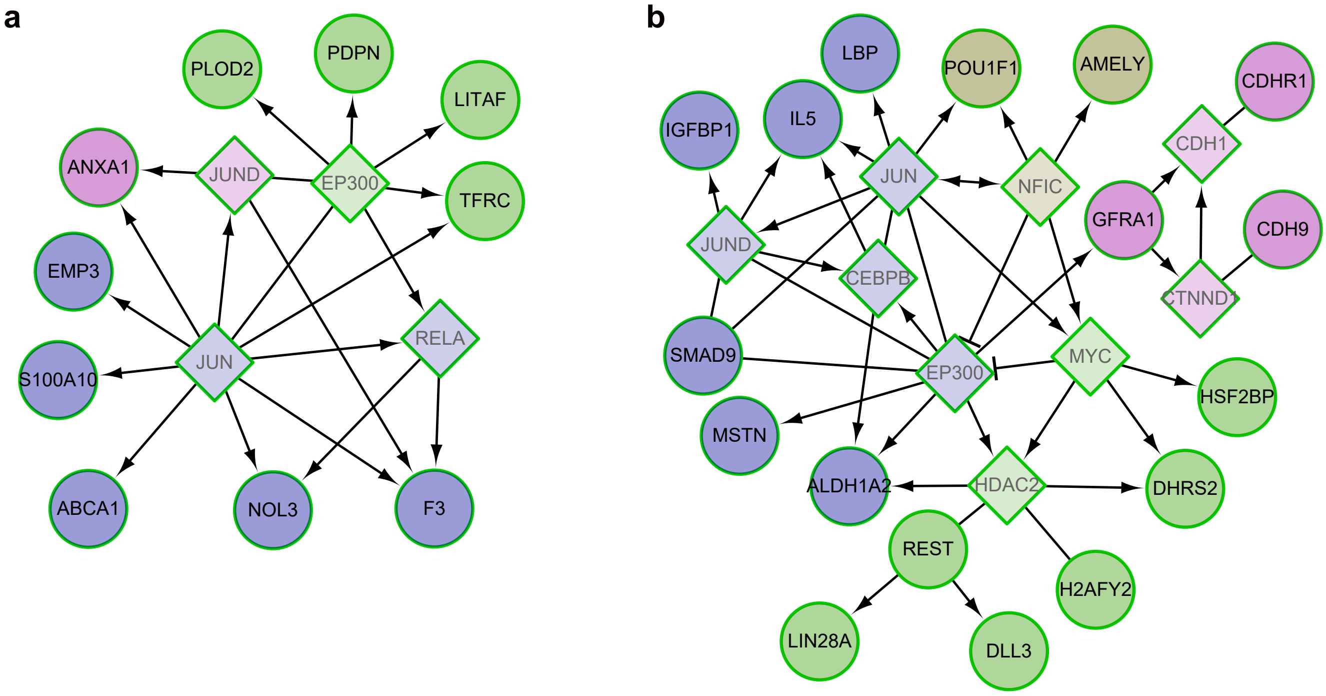

Supplementary Figure S1. Functional interaction networks among PGs. Circle nodes labeled in black represent PGs, and diamond nodes labeled in gray represent linker genes. Nodes in single modules are shown in the same colors. Only nodes with at least one edge are displayed. (**a**) Network for a poor PG set. (**b**) Network for a favorable PG set.

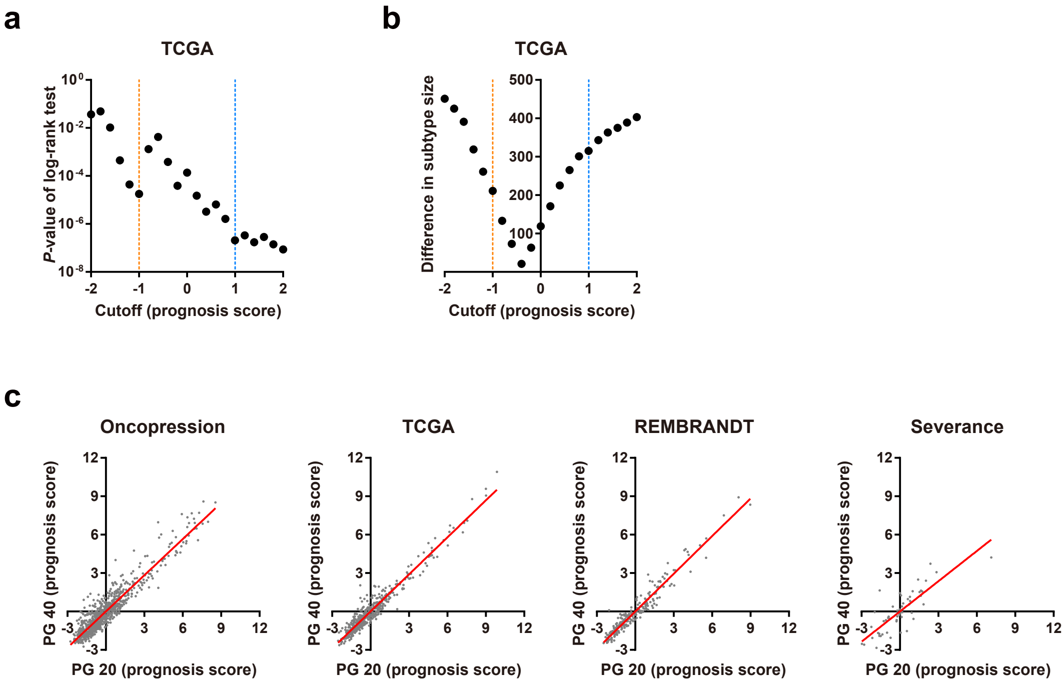

Supplementary Figure S2. Evaluation of cutoff values defining PG sets and prognostic subtypes. (**a** and **b**) *P*-values of log-rank test (**a**) and difference of subtype size (**b**) according to the prognosis score cutoff values for subtype assignment. (**c**) Correlation between prognosis scores obtained from two PG sets (including 40×2 genes and 20×2 genes, respectively).

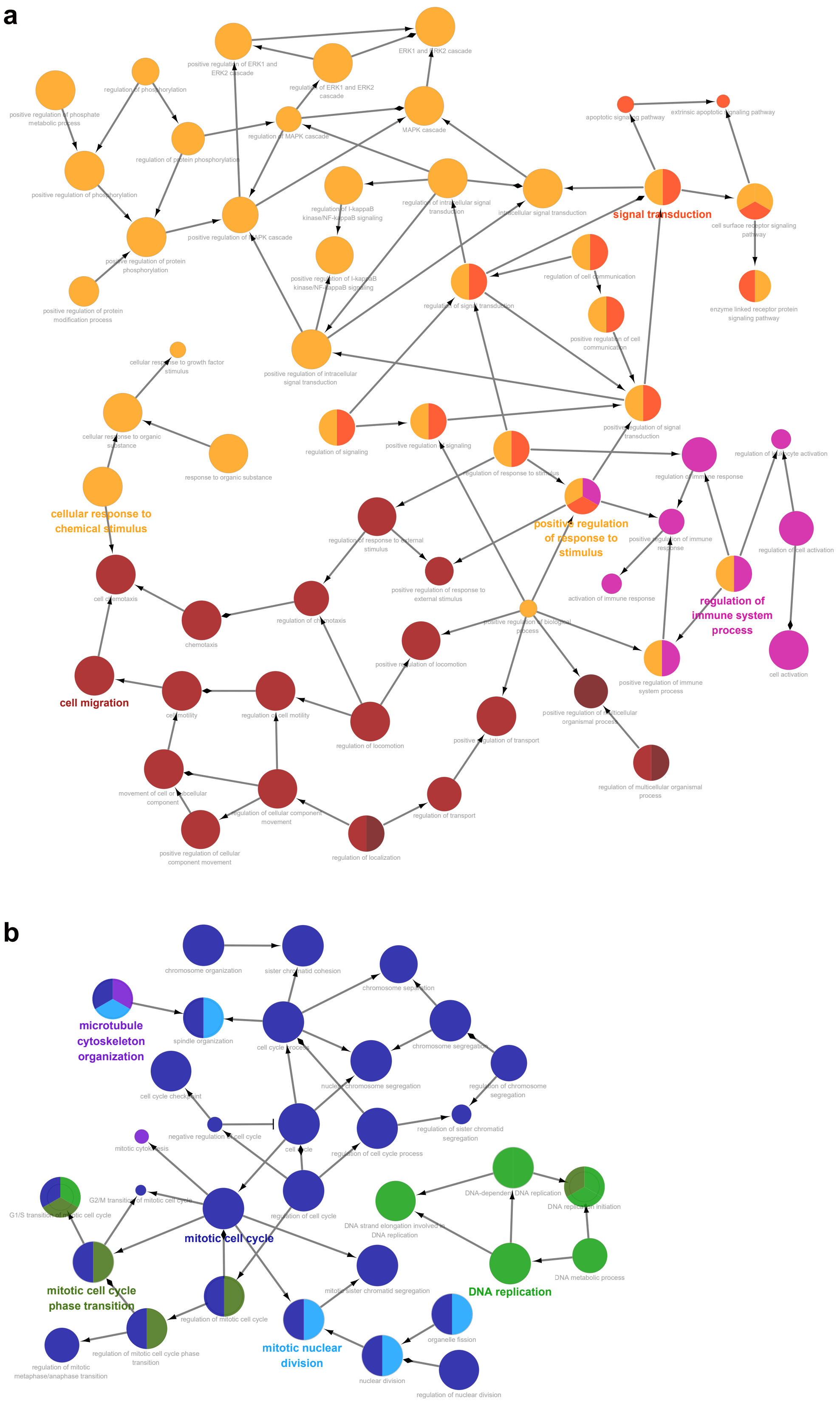

Supplementary Figure S3. Enrichment map of DEGs. DEGs defined in Fig. 4 were functionally annotated, clustered, and visualized as enrichment maps. Each node represents a GO term, with the node size reflecting statistical significance for over-representation. An edge between two nodes denotes GO hierarchy. Node colors reflect clustered modules; the most significant GO terms for each module have highlighted labels. Enrichment map for DEGs upregulated in the poor group (**a**) and in the favorable group (**b**), respectively.

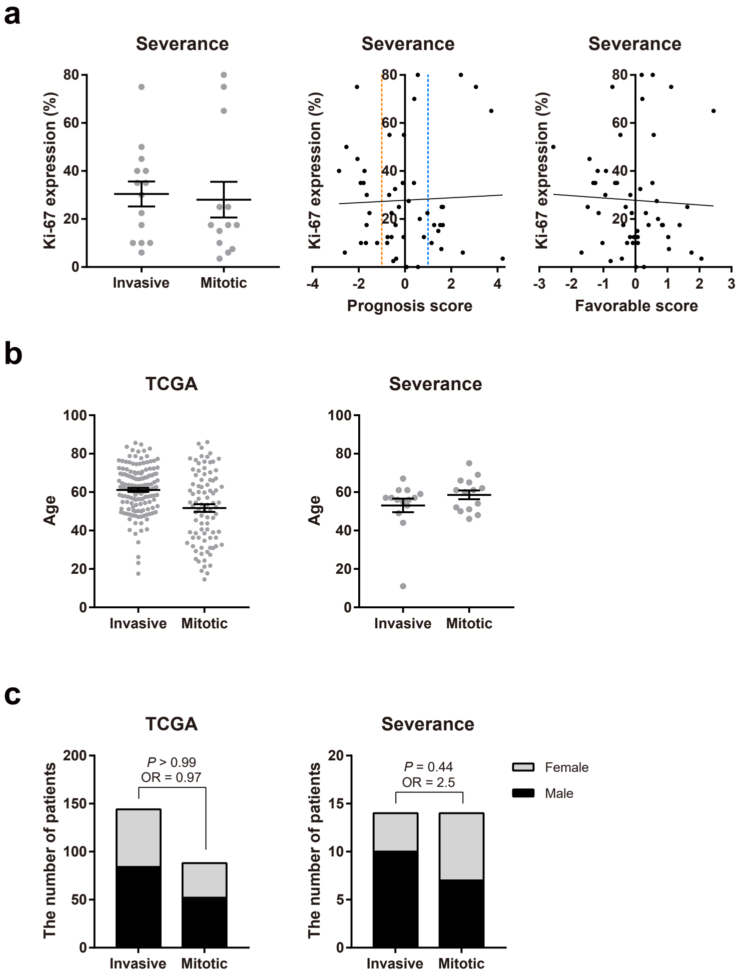

Supplementary Figure S4. Clinical information of GBM patients. (**a**) Expression level of Ki-67 was evaluated in GBM patients (Severance). The scatter plot shows the correlation between Ki-67 expression and prognosis score or favorable score. (**b** and **c**) Comparison of age (**b**) and sex (**c**) in prognostic subtypes of the TCGA-GBM and Severance datasets (OR = odds ratio).

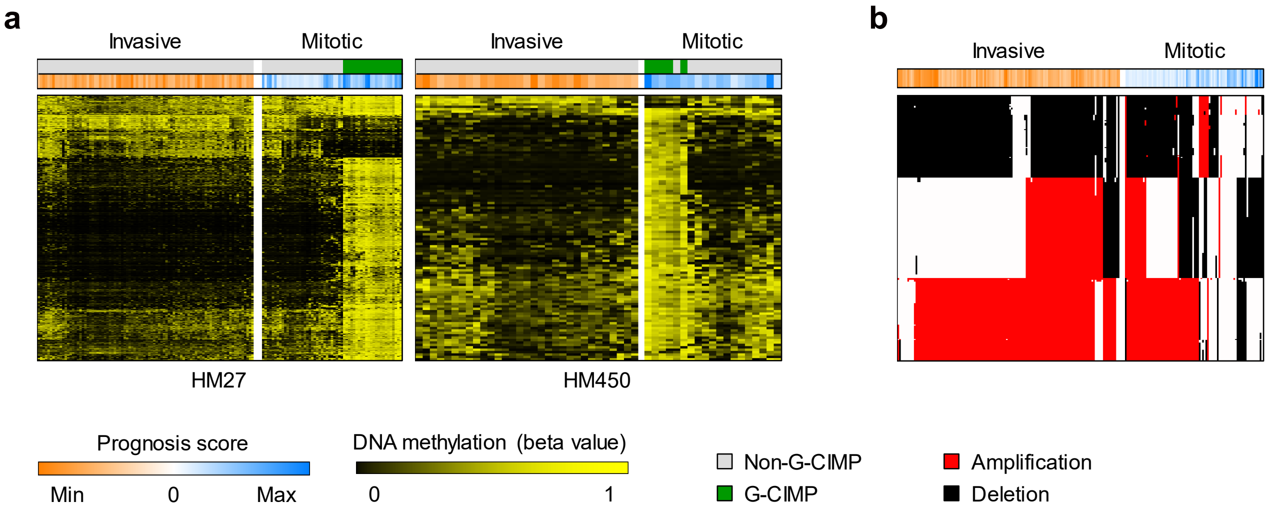

Supplementary Figure S5. Multi-omics signature of prognostic subtypes (TCGA-GBM). (**a**) Distribution of DNA methylation status in promoter regions. Only differentially methylated genes between invasive and mitotic subtypes are shown, overlaid by G-CIMP status. (**b**) Differential CNA between invasive and mitotic subtypes presented as a heat map.

Supplementary Table S1. Risk table (the number of patients) of Kaplan-Meier plots in Fig. 2b.

Oncopression				TCGA				REMBRANDT				Severance			
**OS**	**P**	**F**	**I**	**OS**	**P**	**F**	**I**	**OS**	**P**	**F**	**I**	**OS**	**P**	**F**	**I**
0	35	45	94	0	149	90	256	0	60	49	78	0	16	14	22
2.90	28	44	88	2.83	129	88	229	4.4	55	48	72	4.44	15	14	21
5.95	23	41	80	4.93	106	82	202	7	46	45	64	6.15	14	13	18
8.68	15	41	68	7.56	89	78	176	10	38	43	57	8.52	12	12	15
10.59	12	39	56	10.28	73	71	147	13.3	30	41	46	10.76	10	11	12
13.22	10	36	45	12.98	47	61	122	17.2	27	37	31	12.34	9	10	10
15.12	8	30	35	15.90	38	47	85	20.7	18	30	25	14.84	8	9	7
17.07	8	23	22	19.71	26	40	59	26.4	11	25	18	18.59	5	8	5
23.29	5	17	15	25.03	15	28	38	36	6	21	11	27.89	2	7	4
33.62	2	10	8	35.09	6	17	18	55.2	4	7	5	34.47	2	4	2
88.86	0	1	0	127.5	0	0	1	120.5	0	1	0	51.61	0	1	0

Supplementary Table S2. HR with 95% CI of each covariate calculated by Cox regression model.

	TCGA		Severance	
	**Univariate**	**Multivariate**	**Univariate**	**Multivariate**
**PS**	0.804 (0.736-0.878)	0.841 (0.767-0.922)	0.780 (0.650-0.934)	0.742 (0.605-0.910)
**Age**	1.031 (1.021-1.041)	1.026 (1.015-1.037)	1.016 (0.977-1.056)	1.046 (0.997-1.098)
**Sex**	0.631 (0.484-0.823)	0.634 (0.484-0.831)	1.268 (0.644-2.500)	1.058 (0.524-2.136)
**MGMT**	0.645 (0.500-0.833)	0.751 (0.580-0.973)	0.553 (0.265-1.156)	0.559 (0.253-1.238)

Supplementary Table S3. PG sets and their PCC scores.

Poor (invasive)				Favorable (mitotic)			
**Symbol**	**PCC score**	**Symbol**	**PCC score**	**Symbol**	**PCC score**	**Symbol**	**PCC score**
MSN	-0.0871	SERPINB6	-0.0443	DHRS2	0.0393	LUZP2	0.0101
DYNLT3	-0.0725	DRG2	-0.0439	LBP	0.0196	SMAD9	0.0097
LGALS8	-0.0641	CBR1	-0.0439	HPR	0.0191	AKAP3	0.0095
S100A10	-0.0588	GPRASP1	-0.0436	TMEM100	0.0136	IGFBP1	0.0094
LGALS3	-0.0584	PDPN	-0.0431	H2AFY2	0.0134	RFXAP	0.0092
LRRFIP1	-0.0580	SDF4	-0.0430	TRIM48	0.0133	POU1F1	0.0088
DCTD	-0.0551	FRMD4B	-0.0430	P2RY14	0.0132	AKAP6	0.0086
TMBIM1	-0.0533	GSN	-0.0422	SEC61A2	0.0130	CDHR1	0.0085
EMP3	-0.0533	ABCA1	-0.0414	DACH1	0.0130	REST	0.0081
LITAF	-0.0527	WDR1	-0.0410	MSTN	0.0129	TAT	0.0081
TFRC	-0.0524	DNAJC10	-0.0406	HIST1H4B	0.0121	DDX6	0.0080
CYB561	-0.0521	LOXL1	-0.0405	CDH9	0.0118	SSX3	0.0079
EFEMP2	-0.0512	DNTTIP2	-0.0400	NDUFA13	0.0111	ZP2	0.0078
F3	-0.0503	ANXA1	-0.0400	GFRA1	0.0108	F5	0.0077
TP73-AS1	-0.0500	KHNYN	-0.0400	DLL3	0.0108	SGCG	0.0076
SLC12A7	-0.0488	ARL4C	-0.0396	HSF2BP	0.0108	SLC35E2	0.0076
NOL3	-0.0477	TMF1	-0.0391	ALDH1A2	0.0107	TP53TG5	0.0075
PLOD2	-0.0470	CPQ	-0.0380	CPB1	0.0106	PDZD7	0.0075
OSBPL9	-0.0463	DIRAS3	-0.0375	THNSL1	0.0105	LIN28A	0.0074
TRIP4	-0.0462	S100A11	-0.0371	IL5	0.0102	AMELY	0.0073
